# The therapeutic mechanism of *Chebulae Fructus* in the treatment of immunosuppression in Chinese yellow quail on the basis of network pharmacology

**DOI:** 10.3389/fvets.2023.1123449

**Published:** 2023-05-19

**Authors:** Qiang Wu, Min He, Jing Wang, TieJin Tong, Dan Yang, Huaqiao Tang

**Affiliations:** ^1^Agricultural College, Yibin Vocational and Technical College, Yibin, China; ^2^College of Veterinary Medicine, Sichuan Agricultural University, Chengdu, China

**Keywords:** immunosuppression, *Chebulae Fructus*, network pharmacology, GATA3, TBX21

## Abstract

**Introduction:**

*Chebulae Fructus* (*Terminalia chebula* Retz.) is a well-known traditional Chinese medicine (TCM), one of the family Combretaceae, whose immature fruit is called Fructus Chebulae Immaturus or Zangqingguo. This present study aimed at detecting the target and therapeutic mechanism of Chebulae Fructus against immunosuppression through network analysis and experimental validation.

**Methods:**

Effective components and potential targets of *Chebulae Fructus* were Search and filtered through the Chinese herbal medicine pharmacology data and analysis platform. A variety of known disease target databases were employed to screen the therapeutic target proteins against immunosuppression and thus constructing a protein-protein interaction network. Hub genes and key pathways in this study were identified by continuous project enrichment analysis. Further, the core targets and therapeutic mechanism of *Chebulae Fructus* against immunosuppression in Chinese yellow quail through animal experiment.

**Results:**

Seventy-five identifiable major candidate targets of *Chebulae Fructus* were found and thus constructing a drug-compound-target-disease network. Targets derived from gene enrichment analysis play pivotal roles in lipid and atherosclerosis, fluid shear stress and atherosclerosis, and the hepatitis B pathway. Height of plicate and areas of lymphoid follicle were both increased and the expression of GATA-3 and T-bet was upregulated in Chinese yellow quail fed with *Chebulae Fructus* in animal experiment.

**Conclusion:**

*Chebulae Fructus* may be a helpful Chinese medicine with immunosuppressive effect and prospective applications in future. Further research is also needed to understand the mechanisms of immunosuppression and the mechanism of action of immunomodulators.

## 1. Introduction

Immunosuppression is the most common pathological state induced by stress, anomalotrophy, environmental pollution, and infection (1). It is a common, invisible, and epidemiological disease worldwide, resulting in immunity decline or loss, which was shown to increase the susceptibility of animals to disease and reduce therapeutic effects (2, 3). Under the current conditions of intensive breeding, changes in animal growth patterns, unscientific feeding practices, and drug abuse have led to the frequent occurrence of immunosuppressive diseases (4). Immunosuppression is increasingly common in poultry farming and is difficult to treat. Quail farming is the third largest poultry farming industry after chicken and duck, and Chinese Yellow Quail (*Coturnix japonica*) is one of excellent varieties of quails. It is easy to raise, high in yield and short in alternate generations. However, immunosuppression also occurs on it. No effective pharmacological therapy for immunosuppression has been developed, but some medicinal plants have demonstrated potential application value (5, 6).

Traditional Chinese medicine (TCM) has contributed greatly to the treatment and prevention of diseases in humans (7). In clinical applications, TCM often involves the use of complex components, and its pharmacological effect is reflected in the synergistic effect of multiple targets and multiple pathways and shows obvious advantages in the treatment of complex diseases in animals (8). TCM including the “thunder god” vine (9), Cordyceps sinensis (10), and astragalus has been widely used as alternative treatments for immunosuppression. *Chebulae Fructus* (*Terminalia chebula* Retz.), one of the family Combretaceae, whose immature fruit is called Fructus Chebulae Immaturus or Zangqingguo. And its mature fruit Fructus Chebulae, also called Helile, was firstly written in the “Synopsis of Golden Chamber.” and can be used as a treatment for laryngitis, bacillary dysentery, and tonsillitis. According to the “National Herbal Compendium,” it can treat diarrhea, arrest bleeding, restrain lungs, and resolve phlegm. And the chronic enteritis, chronic bronchitis, asthma, chronic laryngitis, ulcers, hemafecia, and rectocele also can be treated by the Fructus Chebulae (11).

Accordingly, for effective prevention and/or treatment strategies against immunosuppression, detecting the target of *Chebulae Fructus* in treating immunosuppression has become increasingly necessary. The study of systemic regulation in TCM is similar to network pharmacology that combines systems biology, pharmacokinetics and pharmacodynamic properties in order to study drugs, protein targets and their pharmacological activity (12). Drug mechanisms thus can be discovered by networks constructed by combining ‘-omics’ (such as genomics, proteomics, transcriptomics, and metabolomics) profiles and metabolites (13). Drug-gene-target-disease interaction networks were used by network pharmacology to examine the effects of drugs on some diseases, which was similar with TCM theory. It believes that diseases should be diagnosed and treated from a holistic perspective and Chinese medicinal agents and its compounds should be used synergistically (14). In order to define the synergistic effect and mechanism of *Chebulae Fructus*, network pharmacology was used in our research to simulate the network relevance between the active constituent of *Chebulae Fructus* and their targets. An animal experiment was also conducted to verify the effect of the targets screened using network pharmacology on the immunosuppressive effect mediated by *Chebulae Fructus*.

## 2. Materials and methods

### 2.1. Compound profiling and disease target identification

The active constituents of *Chebulae Fructus* were firstly gathered from the Traditional Chinese Medicine Systems Pharmacology (TCMSP) database, Traditional Chinese Medicine Integrated Database (TCMID) [7] Herbal Ingredients Targets Database Introduction (HIT), and other reports (15) After that, the quail target nodes corresponding to the active constituents screened from the Pharmmapper database and the PubMed database were normalized in UniProt.^1^ Then the target nodes related with disease were tapped from the Genecards^2^ database. Later, the whole disease gene targets in this study were standardize with R software and the Bioconductor package after redundancies were deleted (13).

### 2.2. Network establishment

According to the method in the research of Wu (7), the drug-disease crossover genes were performed in our study. Target points related with drugs and diseases were both prepared and crossover target points were filtrated with Venn diagram package and R software. Intersecting protein–protein interactions (PPIs) were analyzed by the Stringdb database,^3^ while the common target points were counted with R software. Cytoscape 3.6.1 software was finally run. And then a drug-compound-target-disease network was created.

### 2.3. Bioinformatic annotation

The R software and Bioconductor package, which includes PPI analysis, (see foornote 3) the gene ontology (GO) annotation database website,^4^ and Kyoto Encyclopedia of Genes and Genomes (KEGG) pathway enrichment analysis,^5^ were used to assess the proteins with overlapping expression patterns through bioinformatics annotation according to the method in the research of Wu (7).

### 2.4. Extract preparation

*Chebulae Fructus* was purchased from the Chengdu Hehuachi Chinese Herbal Medicine Market (Sichuan province), and identified by a TCM scientist at Sichuan Agricultural University. The voucher specimen with number SCP015 was stored in a laboratory for pharmacognosy. *Chebulae Fructus* (100 g) powder was soaked in 2000 mL of distilled water overnight and filtered through 3 pieces of gauze after boiling for 2 h. The filtered liquor was stored at 4°C. The above residue was extracted and filtered once more. The filtrate was concentrated by rotary evaporation to contain 1.0 g/mL of the original medicinal materials at 60 ~ 70°C, and stored at 4°C for future use.

### 2.5. Animal experiment

One hundred Chinese yellow quails (21 days old, half male and half female) were purchased from the Daan Quail farm (Chengdu, Sichuan), and randomly divided into 5 groups (*n* = 20), including the control group, model group, low-dose group (1 g/kg), mid-dose group (2 g/kg), and high-dose group (4 g/kg). Immunosuppression model was established by intramuscular injection of 80 mg/kg of cyclophosphamide (Shanghai Yuanye Biotechnology Co., Ltd.) from day 1 to day 3 (16, 17). The control group was treated with an equivalent amount of normal saline. Afterward, *Chebulae Fructus* extract was administered by gavage from days 4 to 10. Dosing volumes were adjusted using normal saline to deliver equivalent amounts. During the trial, the birds had free access to laboratory feed and water. This animal experiment was conducted obedience to the administration of Affairs Concerning Experimental Animals of the State Council of the People’s Republic of China and its protocol was authorized by the Committee on Experimental Animal Management of Yibin Polytechnic College in Sichuan (protocol no. 2016051407).

### 2.6. Pathological examination

At the end of the experiment, feeding was stopped for 12 h and three of them were randomly selected, anesthetized and euthanized. The bursa of Fabricius was moved and stored in liquid nitrogen. Another part of the bursa of Fabricius was fixed in 4% paraformaldehyde for making paraffin-embedded tissue sections and performing hematoxylin and eosin (H.E.) staining. The structural changes in the bursa of Fabricius were observed by 400x microscopy, and the height of the plicate and lymphoid follicle areas of the bursa of Fabricius was measured by 100x microscopy equipped with Image-Pro Plus 6.0 software (Boao Yijie (Beijing) Technology Co., Ltd.).

### 2.7. The expression of *GATA-3* and *T-Bet*

Primers were designed for the *β-actin*, *GATA-3*, and *T-bet* genes based on the gene sequences downloaded from the National Center for Biotechnology Information (NCBI; Table 1). RNA was extracted from the bursa of Fabricius tissues that were frozen and ground into powder using liquid nitrogen. The expression of *GATA-3*, and *T-bet* was detected by fluorescence quantitation polymerase chain reaction (PCR) analysis of cDNA, which was constructed from the above-prepared RNA using the Prime-Script TMRT Kit.

**Table 1:** Primer sequences and amplification lengths.

Name	Sequence	Length (bp)
*β-actin-F*	GCGTGACATCAAGGAGAAGC	190
*β-actin-R*	CACAGGACTCCATACCCAAGAA	
*GATA-F*	ACTACTTGTGTAACGCCTGTGGAC	132
*GATA-R*	GTGGTGGTGGTCTGACAGTTAGC	
*T-bet-F*	CCGACTCACCCAACACC	247
*T-bet-R*	GTAAGCAGTGACAGCAATGAA	

### 2.8. Data analysis

This analysis was performed with PASW Statistics 22.0 software package (ICM, Armonk, NY, United States). Data were expressed as the means and standard deviation (means ± SD), and comparison between groups was performed by the independent-sample *t*-test.

## 3. Results

### 3.1. The assumed targets of *Chebulae Fructus* and immunosuppression

Ninety-six *Chebulae Fructus* compounds (Supplementary Table S1) were collected from the TCMSP database^6^ and analyzed by a network for its different targets number of each compound and significant target overlapped. Then seventy-five target proteins from a total of 108 target points were found by UniProt.^7^

The effectiveness of *Chebulae Fructus* in preventing and controlling immunosuppression were decided by the synergy between multiple compounds and their target points. The target points related with immunosuppression from the GeneCards and OMIM databases were collected for distinguishing disease target. After screening key nodes for removing duplicates, totally 3,573 target points were found by the topological analysis of protein interaction network nodes.

### 3.2. Network analysis of targets

**Figure 1:**
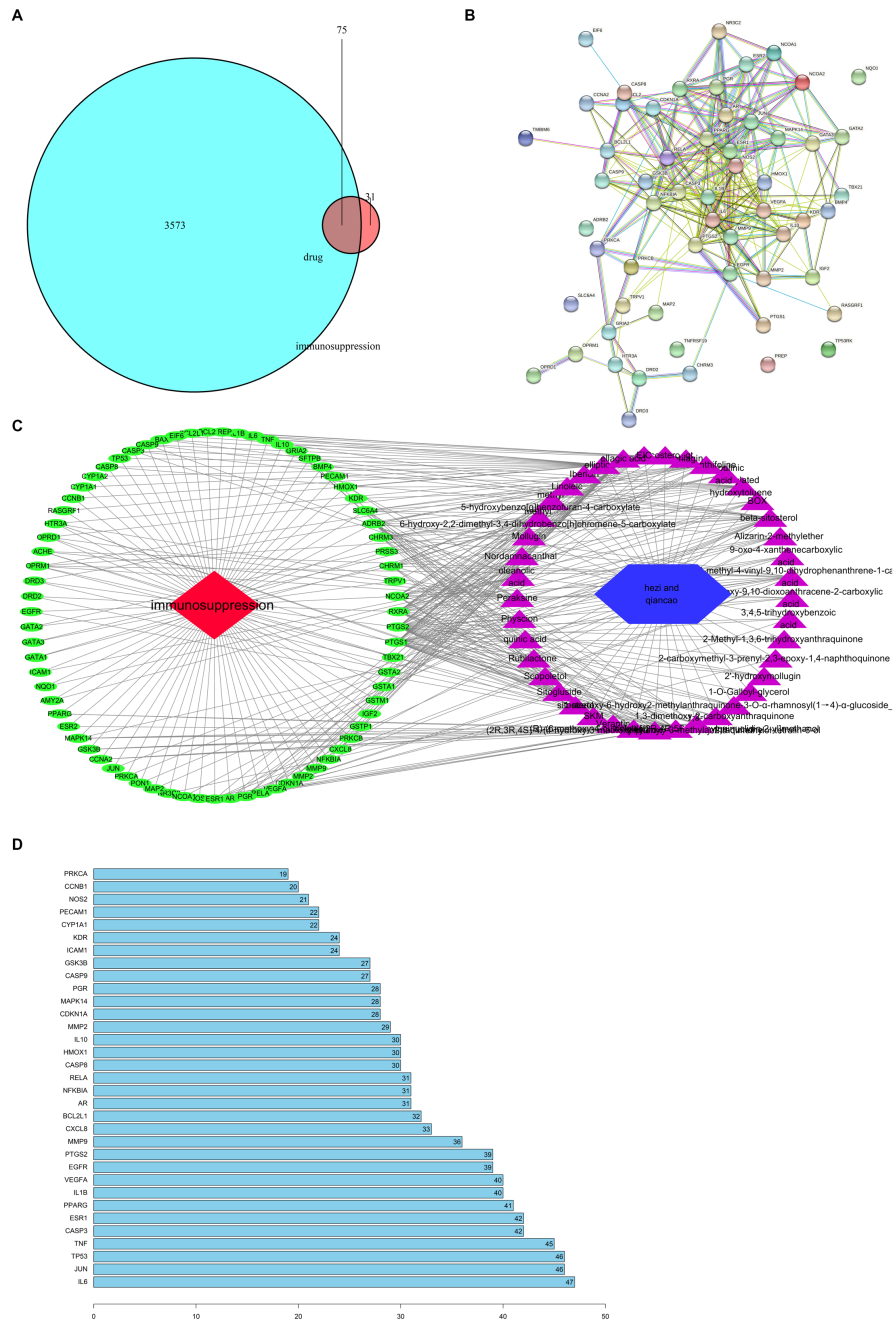
Network analysis of targets. **(A)** Venn diagram of drug and disease targets; and **(B)** PPI network; and **(C)** ‘drug-compound-target-disease’ network. Blue indicates drug-disease intersection targets. Red indicates immunosuppression, and other colors indicate active compounds; and **(D)** Core gene.

Target points related with drug and disease were listed as 2 independent sets with the R software analysis. A Venn diagram representing the set and its relationship in the form of a closed loop with the fixed position was shown in Figure 1A. Totally 75 interaction target points were obtained in this analysis and 75 simulated target points and 720 edges were got by String-db, (see footnote 3) which was shown in Figure 1B.

An interactive drug-compound-target-disease network was also created in Figure 1C. *T-bet*, whose alias is *TBX21*, can be an immunosuppression-related gene affected by *Chebulae Fructus*. Multiple ingredients promoted the expression of immunosuppression-related genes including *IL6*, *JUN*, *DTP 3*, and *CAP* (Figure 1D).

### 3.3. Predicting functional enrichment analysis of *Chebulae Fructus*

**Figure 2:**
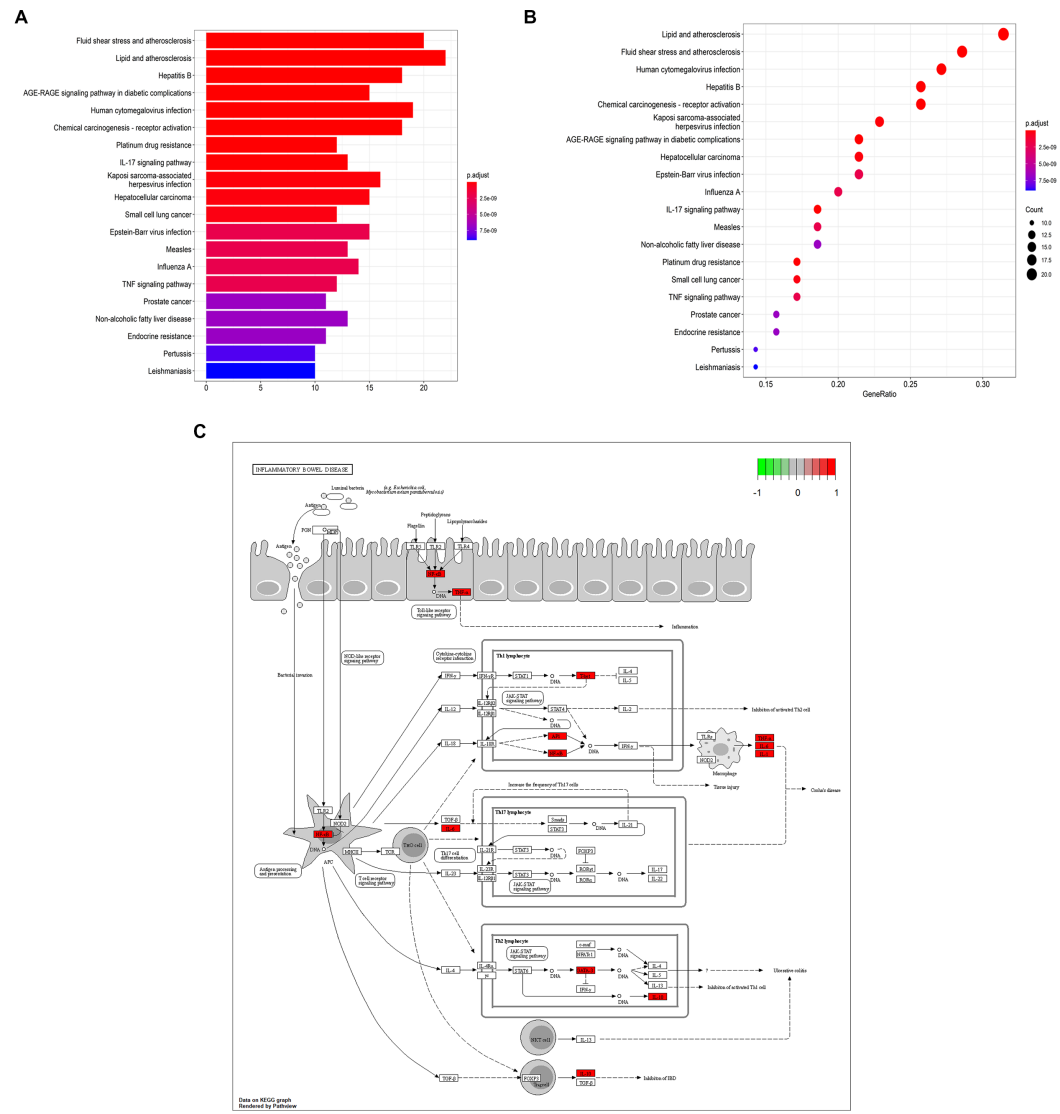
Bioinformatic analyses of drug-disease intersection proteins. **(A)** Gene ontology annotations; **(B)** KEGG enrichment analysis; **(C)** *T-bet* and *GATA3* in Inflammatory bowel disease.

GO annotation showed the expressed drug-disease crossover targets mainly relating with DNA-binding transcription factor binding, RNA polymerase II-specific DNA-binding, transcription factor binding, peptide binding, and amide binding (Figure 2A). The related genes were *RELA*, *TBX21,* and *GATA3* (Supplementary Table S2).

Moreover, KEGG enrichment analysis revealed that many target genes are closely associated with lipids and atherosclerosis, fluid shear stress and atherosclerosis, human cytomegalovirus infection and hepatitis B (Figure 2B; Supplementary Table S3). The results showed that *Chebulae Fructus* may affect the function of immune cells to treat immunosuppression, and the main target genes of immunosuppression are *T-bet* and *GATA3* (Figure 2C). Some experiments were designed on the basis of molecular mechanisms of these above predictions and the network analysis results to complete validation of hypotheses at the cellular level.

### 3.4. Clinical symptoms

No clinical manifestations such as feeding, drinking, movement and behavioral problems were observed, but all groups of Chinese yellow quail were lethargic while on cyclophosphamide except for the control group. After injecting the drug, the quail were relieved of their lethargy.

### 3.5. Histopathology, height of plica and lymphoid follicle areas

**Figure 3:**
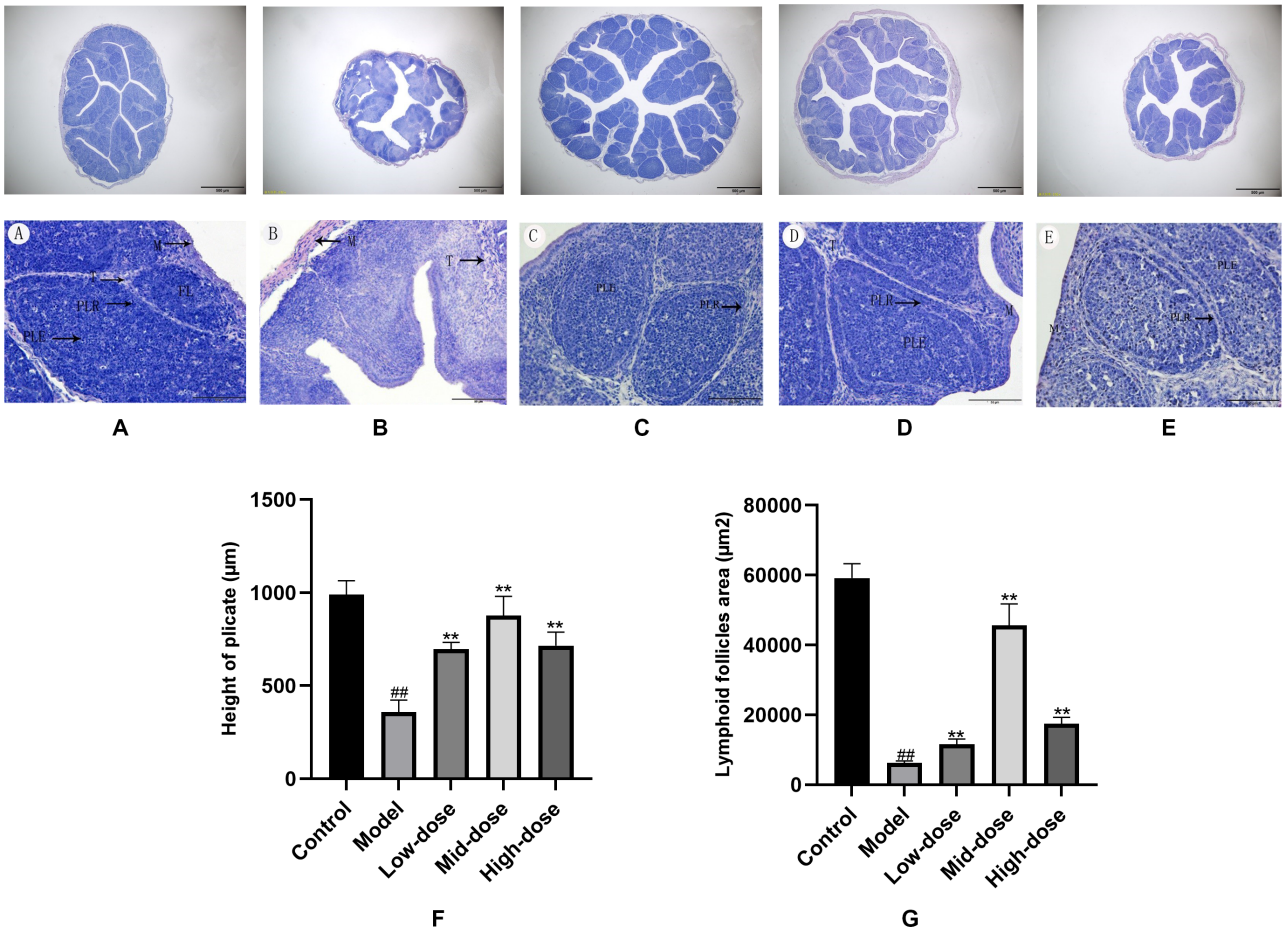
Histological changes of Bursa of Fabricius (H.E. bar = 50 μm) and (H.E. bar = 500 μm). **A** (blank group), **B** (model group), **C** (low-dose group), **D** (mid-dose group) and **E** (high-dose group). **F,G**, Height of plica and lymphoid follicle areas in the bursa of Fabricius. All data were expressed as means ± SD (*n* = 20). ^##^ *p* < 0.05 vs. Control group, ^**^ *p* < 0.01 vs. Model group.

Images of histopathology sections of the bursa of Chinese yellow quail from each group of the trial were shown in Figures 3A–E. All bursal structures were intact and clear, with a clear demarcation between the cortex and medulla. The height of the plicate and lymphoid follicle areas showed a similar trend from high in the control, mid-dose, high-dose, and low-dose groups, to low in the model group. Compared with the control group, the height of the plicate and lymphoid follicle areas of quails in model group were significantly decreased (*p* < 0.01). The results are listed in Figures 3F,G.

### 3.6. Relative expression of *GATA-3* and *T-Bet*

**Figure 4:**
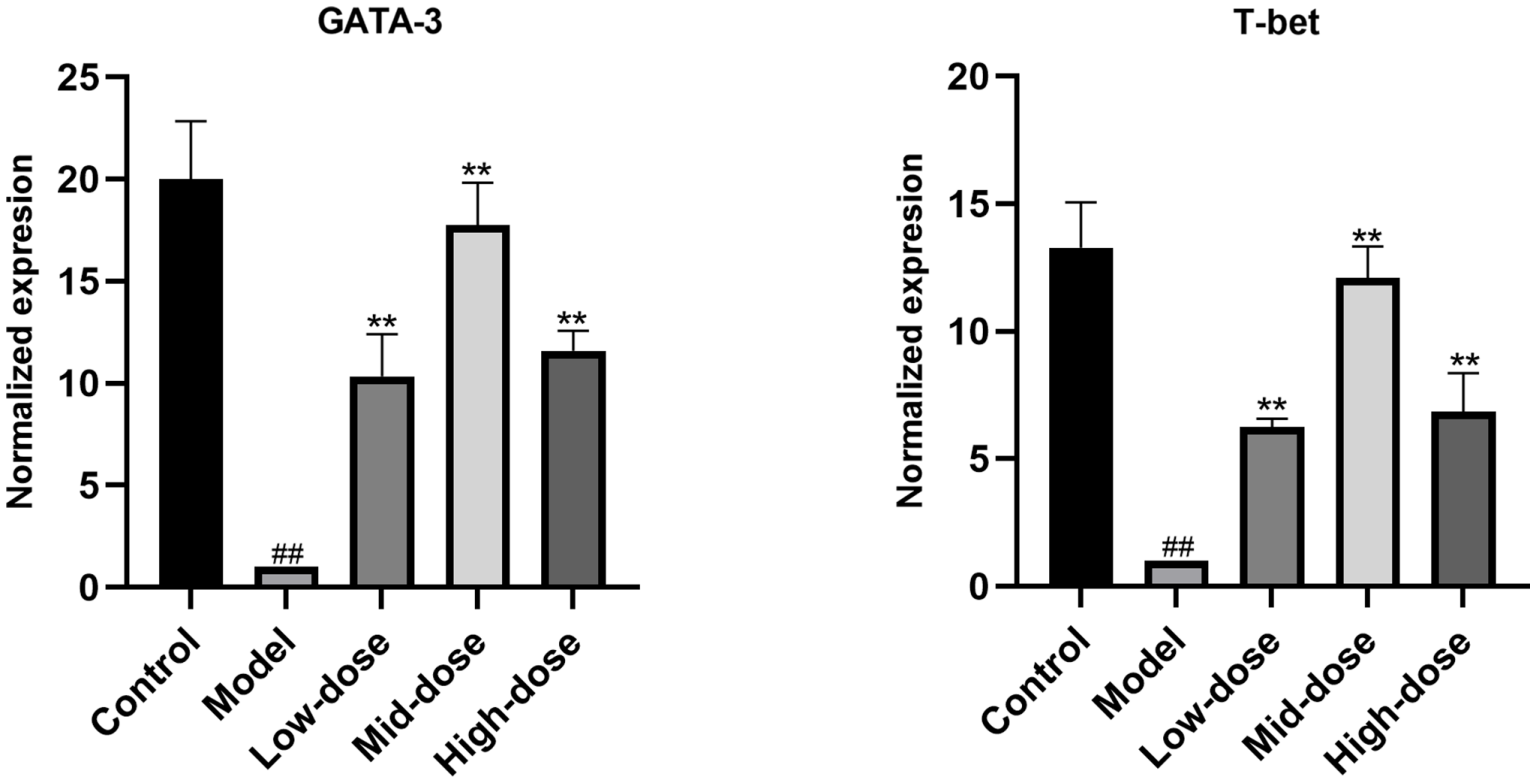
Relative expression of *GATA-3* and *T-bet* in the bursa of Fabricius. All data were expressed as means ± SD (*n* = 20). ^##^ *p* < 0.05 vs. Control group, ^**^ *p* < 0.01 vs. Model group.

As shown in Figure 4, compared with the control group, the relative mRNA expression level of *T-bet* and *GATA-3* in the Model group was downregulated, respectively. Compared with the Model group, *Chebulae Fructus* can upregulated the expression of *T-bet* and *GATA-3* in Chinese yellow quail fed with *Chebulae Fructus* (*p* < 0.01).

## 4. Discussion

The pharmacodynamics of Chinese medicine compounds and their mechanisms of action play vital roles in modernization of TCM (18). Drug effects on the bio-net can be explained by network pharmacology and systems biology from the perspective of macroscopic or overall regulation, which can supply new technical means and research thoughts for study of the mechanisms of Chinese medicinal compounds (18). At present, many studies have shown that the use of network pharmacology to reveal the molecular mechanism of active ingredients of traditional Chinese medicine and their targets in various diseases (19). For example, Dong et al. discovered the potential targets of astragalus membranaceous-angelica sinensis compound acting on diabetic nephropathy by network pharmacology (20). Wei et al. elaborated on the mechanism of Sinisan against non-alcoholic fatty liver disease using network pharmacology (21). Li et al. identified the intergenes of niacin and COVID-19 as potential therapeutic targets based on network pharmacological and bioinformatics analysis (22). Considering the complexity of the active ingredients of *Chebulae Fructus* and the diversity of the potential regulatory targets in Chinese yellow quail, it is necessary to use network pharmacology analysis to screen target points of the compounds in *Chebulae Fructus* and immunosuppression-related target points from the multiple databases. And then, the drug-compound-target-disease network was created to predict the latent target points of *Chebulae Fructus.* Sheng et al. assessed major compontent of aqueous extract of *Chebulae Fructus*, by HPLC-ESI-MS, which mainly contains gallic acid, 3,4,6-tri-O-galloyl-β-d-Glc, corilagin and ellagic acid (23).

A variety of known disease target databases were used to screen anti-immunosuppressive therapeutic target proteins and construct protein–protein interaction networks. From the TCMSP database, network pharmacological analysis of *Chebulae Fructus* identified 96 compounds, and 3,573 target gene-regulated major pathways related to immunosuppression. RWR analysis also identified key genes closely related to the targets of *Chebulae Fructus*, and various components promoted the expression of immunosuppression-related genes, including IL6, JUN, DTP 3, and CAP. In particular, 3 key genes, *RELA*, *TBX21*, and *GATA3*, were regulated by 3 or more components of *Chebulae Fructus* linked to inflammation and intestinal mucosal immunity. Most of the compounds were classified as polyphenols, which have been indicated as the most effective ingredients in *Chebulae Fructu* (17, 24). The immunology community has recognized that naïve CD4 T cells need to make important decisions when they are activated, namely differentiation into Th1, Th2, Th17 (interleukin-17-producing T helper cells), follicular T helper cells, or regulatory T cells to coordinate various adaptive immune responses. The main molecular basis of Th1/Th2 effector fate selection was preliminarily determined by using an excellent reducing agent *in vitro* culture system, through which transcription factors *T-bet* and *GATA3* were identified as the main regulators of Th1 and Th2 cell differentiation, respectively (25). These results suggest that potential therapeutic targets of *Chebulae Fructus* are important for immunosuppressive therapy. The KEGG enrichment analysis revealed that a lot of target genes strongly related with lipid and atherosclerosis, fluid shear stress and atherosclerosis, human cytomegalovirus infection, and hepatitis B. Intestinal mucosal damage is one of the main causes of immunosuppression, and the target genes leading to immunosuppression were mainly *T-bet* and *GATA3*, which may be related to the immune regulation (26–28). The results showed that *Chebulae Fructus* may affect the function of immune cells to treat immunosuppression, and the main target genes of immunosuppression are *T-bet* and *GATA3*.Studies have reported that the extract of *Chebulae Fructus* significantly inhibits the growth of breast cancer cells and lung cancer cells (29). Saleem et al. used ethanol extract of *Chebulae Fructus* to treat liver cancer, breast cancer, osteosarcoma, prostate cancer cell lines (30), and confirmed that can be used for clinical treatment of HCC (31), however, its clinical role in the treatment of yellow quail immunosuppression targets and pathways need further research.

The quail (Coturnix) is an economically important poultry because of its high meat quality and nutritious eggs. In addition, quail maturation, high egg production rate, short spawning interval, rapid growth, limited feed and space required, and fast return on investment have made quail farming the third largest poultry industry in some Asian countries after chickens and ducks (32, 33). In addition, quail is an important laboratory research animal that is widely used for developmental biology and toxicology testing (34–36). Wang et al. proved that Lead induced thymic immunosuppression in Japanese quail (*Coturnix japonica*) via oxidative stress-based T cell receptor pathway signaling inhibition (37). Studies have reported that traditional Chinese medicine can improve the production performance, enhance immunity and relieve oxidative stress of quai, such as ellagic acid (38), quercetin (39), *Yucca schidigera* extract (40). We confirmed that Myrobalan could treat cyclophosphamide-induced immunosuppression in Chinese yellow quail by validating the key therapeutic targets screened using network pharmacology. Cyclophosphamide is a common immunosuppressant, which can destroy the DNA of normal cells, reduce the number of white blood cells by inhibiting hematopoiesis, and inhibit the growth of the bursa of Fabricius and division of B lymphocytes (41). He et al. established a cyclophosphamide-induced immunosuppressive pathological model of Chinese yellow quail and evaluated the successful establishment of the experimental immunosuppressive model by recording the changes of spleen organ index and tissue structure of Chinese yellow quail before and after cyclophosphamide injection (16). We analyzed the effects of cyclophosphamide on the histopathology, fold height and lymphatic follicles of the bursa structure of Chinese yellow quail. Pathological results showed that cyclophosphamide injection reduced the height of the plicate and lymphoid follicle areas of the bursa of Chinese yellow quail, indicating the establishment of an immunosuppression model was successful. There was no difference in the height of the plicate and lymphoid follicle areas between the *Chebulae Fructus*-fed group and the control group, indicating that *Chebulae Fructus* could recover quail from immunosuppression, even may help to enhance the immunity of poultry and improve the body’s resistance.

In the immune response, various immune cells, including T and B lymphocytes, macrophages, and dendritic cells, are activated and tightly bound to fight unwanted and foreign factors. In these cells, lymphocytes play a vital role in host defense as part of the innate and adaptive immune system. T lymphocytes are closely involved in cell-mediated immune responses by differentiating into effector T cells, also known as CD8+ cytotoxic T cells (Tc) and CD4+ helper T cells (Th cells). CD4+ T cells differentiate into two main subtypes, Th1 and Th2, based on the lymphokines they produce. Th1 cells produce interferon (IFN)-c and interleukin (IL)-2 as their signature cytokines, which act as pro-inflammatory cytokines, while Th2 cells secrete anti-inflammatory cytokines, such as *IL-4* and *IL-10*, which regulate their own damage to pro-inflammatory cytokines. Thus, Th1 cells generally promote inflammation and tumor immunity, while Th2 cells promote B-cell-mediated humoral immunity against extracellular pathogens. The transcription factors *T-bet* and *GATA-3* are two intracellular molecules that are specifically expressed in Th1 and Th2 cells, respectively, and ultimately determine the differentiation of Th0 to Th1/Th2 (42). Transcription factors *GATA-3* and *T-bet* are the main regulators of Th2 and Th1 differentiation, respectively, and promote the development of Th cells. The expression of T-bet and *GATA-3* affects the steady-state stability of Th1/Th2, and the ratio of *T-bet/GATA-3* can reflect the state of Th1/Th2 (43–45). Therefore, we evaluated the expression levels of *GATA-3* and *T-bet* mRNA and protein in bursa of Fabricius (45). The gene expression results showed that the immune response in the *Chebulae Fructus* -fed group did not differ from the control group, which corresponded to the pathological results and indicated that *Chebulae Fructus* could recover quail from immunosuppression. These findings demonstrated that *Chebulae Fructus* had an important treatment value in relieving immunosuppression. The structure of the bursa of Chinese yellow quail treated with *Chebulae Fructus* was complete, and the number of lymphocytes was significantly increased. The network pharmacology results showed that polyphenols were the main ingredient in repairing bursa structures and promoting immunity (46), such as green tea polyphenols (47), fruit polyphenols (48), resveratrol (49) etc. This is a bioinformatics-based study that also predicts the binding site of the active ingredient of a drug to a target gene through molecular docking simulations. Although animal experiments have been introduced, there is not enough content related to them, and other experiments are needed to examine the effects of *Chebulae Fructus* on immunosuppression. For example, the expression of *IL6*, *JUN*, DTP 3, and *CAP* in immune organs should be detected. It is still of great necessity to verify the potential mechanism of *Chebulae Fructus* in the treatment of immunosuppression in Chinese yellow quai in combination with more cell experiments and animal experiments.

## 5. Conclusion

In this research, the targets and therapeutic mechanism of *Chebulae Fructus* against immunosuppression in Chinese yellow quail preliminarily analyzed by means of network pharmacology. In network visualization, the core genes of *RELA*, *TBX21*, and *GATA3* were related to the immunosuppression treatment target of *Chebulae Fructus* can be deduced from a large array of data integrations and calculations. Our animal experiment results showed that *Chebulae Fructus* could relieve cyclophosphamide-induced bursal immunosuppression in Chinese yellow quail and enhance the expression of *GATA-3* and *T-bet*, which are the main transcriptional regulators of immune cells. This study proved *Chebulae Fructus* may be a promising, long-lasting immunosuppressive therapy, also provides a theoretical basis for anti-immunosuppression for the mechanism of *Chebulae Fructus* and its future experimental verification.

## Data availability statement

The original contributions presented in the study are included in the article/Supplementary material, further inquiries can be directed to the corresponding author.

## Ethics statement

The animal study was reviewed and approved by the Committee on Experimental Animal Management of Yibin Polytechnic College in Sichuan (protocol no. 2016051407).

## Author contributions

QW: conceptualization and funding acquisition. MH: methodology. JW, TT, and DY: writing-original draft preparation. QW, HT, and DY: writing-review and editing. QW and HT: supervision. All authors contributed to the article and approved the submitted version.

## Funding

This research was funded by the Key Laboratory of traditional Chinese veterinary medicine of the State Ethnic Affairs Commission ([2020]07) and the Research Foundation for Advanced Talents of Yibin Vocational and Technical College (ybzysc20bk04).

## Conflict of interest

The authors declare that the research was conducted in the absence of any commercial or financial relationships that could be construed as a potential conflict of interest.

## Publisher’s note

All claims expressed in this article are solely those of the authors and do not necessarily represent those of their affiliated organizations, or those of the publisher, the editors and the reviewers. Any product that may be evaluated in this article, or claim that may be made by its manufacturer, is not guaranteed or endorsed by the publisher.

## Footnotes

^1^ https://www.uniprot.org/

^2^ https://www.genecards.org/

^3^ http://string-db.org/

^4^ http://www.geneontology.org

^5^ http://www.genome.jp/kegg/

^6^ https://tcmsp-e.com/

^7^ https://www.uniprot.org/
