# Supplementary material for: The therapeutic mechanism of Chebulae Fructus in the treatment of immunosuppression in Chinese yellow quail on the basis of network pharmacology
Source: Front Vet Sci. 2023 May 19;10:1123449. doi: 10.3389/fvets.2023.1123449 (PMC10235497; doi:10.3389/fvets.2023.1123449)
Supplement: Supplementary file 1 [file Table_1.DOCX]

SUPPLEMENTARY DATA

**Based network pharmacology method to discovered the targets and therapeutic mechanism of *Chebulae Fructus* against immunosuppression in Chinese Yellow Quail**

Qiang Wu^1*^, Min He^2^, Jing Wang^1^, TieJin Tong^1^, Huaqiao Tang^2*^

^1^ Agricultural College, Yibin Vocational and Technical College, Yibin 644000, China;

^2^ Department Pharmacy, Sichuan Agricultural University, Chengdu 611130, China

**^*^** Co-Correspondence: Qiang Wu: wuqiangfirst@163.com; Huaqiao Tang: turtletang@163.com

Table S1 96 compounds from Chebulae Fructus

| ID | Molecule Name | MW |
| --- | --- | --- |
| 1 | EIC | 280.5 |
| 2 | BOX | 121.12 |
| 3 | Sennoside C | 848.82 |
| 4 | Sennoside E_qt | 524.5 |
| 5 | 1-O-Galloyl-glycerol | 244.22 |
| 6 | butylated hydroxytoluene | 220.39 |
| 7 | quinic acid | 191.18 |
| 8 | Ethyl geranate | 196.32 |
| 9 | SKM | 174.17 |
| 10 | corilagin | 634.49 |
| 11 | 3,4,5-trihydroxybenzoic acid | 170.13 |
| 12 | Melissic acid | 452.9 |
| 13 | Glucosol | 472.78 |
| 14 | Maslinic acid | 472.78 |
| 15 | 7-Dehydrosigmasterol | 414.79 |
| 16 | Gallic acid-3-O-(6'-O-galloyl)glucoside | 484.4 |
| 17 | chebulic acid | 356.26 |
| 18 | palmitic acid | 256.48 |
| 19 | Arjunolic acid | 488.78 |
| 20 | Linoleic | 280.5 |
| 21 | α-Santalol | 220.39 |
| 22 | Pentagalloylglucose | 940.72 |
| 23 | 3-Dehydroshikimate | 172.15 |
| 24 | Tannic acid (Corilagin) | 636.51 |
| 25 | D-Altro-3-heptulose | 210.21 |
| 26 | ellipticine | 246.33 |
| 27 | Peraksine | 310.43 |
| 28 | (R)-(6-methoxy-4-quinolyl)-[(2R,4R,5S)-5-vinylquinuclidin-2-yl]methanol | 324.46 |
| 29 | Teresautalic acid | 166.24 |
| 30 | (2Z)-2-[(E)-3-cyano-3-[(2S,3R,4S,5S,6R)-3,4,5-trihydroxy-6-(hydroxymethyl)oxan-2-yl]oxyprop-2-enylidene]butanedioic acid | 359.32 |
| 31 | Triglochinin_qt | 197.16 |
| 32 | (1S,4aR,6aR,6aS,6bR,8aR,9R,10R,11R,12aR,14bS)-1,10,11-trihydroxy-2,2,6a,6b,9,12a-hexamethyl-9-methylol-1,3,4,5,6,6a,7,8,8a,10,11,12,13,14b-tetradecahydropicene-4a-carboxylic acid | 504.78 |
| 33 | beta-Glucogallin | 332.29 |
| 34 | chebulagic acid | 954.7 |
| 35 | chebulinic acid | 956.72 |
| 36 | chebupentol | 490.8 |
| 37 | punicalagin | 1,084.75 |
| 38 | 2,6-Dimethylheptadecane | 268.59 |
| 39 | Catharanthamine | 809.06 |
| 40 | Cheilanthifoline | 325.39 |
| 41 | Oleanolic acid deriv. | 498.82 |
| 42 | oleanolic acid | 456.78 |
| 43 | (2R,3R,4S)-4-(4-hydroxy-3-methoxy-phenyl)-7-methoxy-2,3-dimethylol-tetralin-6-ol | 360.44 |
| 44 | Sitogluside | 576.95 |
| 45 | beta-sitosterol | 414.79 |
| 46 | sitosterol | 414.79 |
| 47 | Tectochinon | 222.25 |
| 48 | Scopoletol | 192.18 |
| 49 | Physcion | 284.28 |
| 50 | Mollugin | 284.33 |
| 51 | daucosterol | 576.95 |
| 52 | daucostero_qt | 414.79 |
| 53 | 1,3,6-trihydroxy-2-methylanthraquinone-3-O-(3'-O-acetyl)-α-rhamnosyl(1→2)-glucoside | 622.58 |
| 54 | 2-Methyl-1,3,6-trihydroxyanthraquinone | 270.25 |
| 55 | 1,3,6-trihydroxy-2-methylanthraquinone-3-O-(6'-O-acetyl)-α-rhamnosyl(1→2)-β-glucoside | 608.55 |
| 56 | 1,3,6-trihydroxy-2-methylanthraquinone-3-O-α-rhamnosyl-(1→2)-β-glucoside | 578.57 |
| 57 | 2-Methyl-1,3,6-trihydroxy-9,10-anthraqui-none-3-O-beta-D-xylosyl-(1-->2)-beta-D-(6'-O-acetyl) glucoside | 606.58 |
| 58 | 1,3-dimethoxy-2-carboxyanthraquinone | 312.29 |
| 59 | 1,3-dihydroxy-2-hydroxymthylanthraquinone-3-O-xylosyl(1→6)-glucoside | 564.54 |
| 60 | 1,3-dihydroxy-2-hydroxymthylanthraquinone-3-O-xylosyl(1→6)-glucoside_qt | 284.28 |
| 61 | Ibericin | 298.31 |
| 62 | 1,3- dihydroxy-3-methylanthraquinone | 254.25 |
| 63 | 1,4-dihydroxy-6-methyl-9,10-anthraquinone | 254.25 |
| 64 | 1-hydroxy-2-methyl-6-methoxyanthraquinone | 268.28 |
| 65 | Danthron methyl derivative | 254.25 |
| 66 | Alizarin-2-methylether | 254.25 |
| 67 | Deoxylucidin | 254.25 |
| 68 | 7-hydroxy-8-methyl-4-vinyl-9,10-dihydrophenanthrene-1-carboxylic acid | 278.32 |
| 69 | 1-acetoxy-6-hydroxy2-methylanthraquinone-3-O-α-rhamnosyl(1→4)-α-glucoside | 616.67 |
| 70 | 1-acetoxy-6-hydroxy2-methylanthraquinone-3-O-α-rhamnosyl(1→4)-α-glucoside_qt | 310.32 |
| 71 | 2-carbamoyl-3-hydroxy-1,4-naphthoquinone | 219.21 |
| 72 | 2'-hydroxymollugin | 302.35 |
| 73 | 2-carboxymethyl-3-prenyl-2,3-epoxy-1,4-naphthoquinone | 286.3 |
| 74 | 4-hydroxy-9,10-dioxoanthracene-2-carboxylic acid | 266.21 |
| 75 | 6-methoxygeniposidic acid | 240.28 |
| 76 | Verantin | 256.22 |
| 77 | Rubiadin | 254.25 |
| 78 | Rubianic acid | 534.51 |
| 79 | Alizarin | 240.22 |
| 80 | 2-Methyl-1,4-dihydroxyanthraquinone | 254.25 |
| 81 | Nordamnacanthal | 268.23 |
| 82 | 1-Hydroxy-2-methylanthraquinone | 238.25 |
| 83 | Pallasone | 374.62 |
| 84 | methyl 5-hydroxybenzo[g]benzofuran-4-carboxylate | 242.24 |
| 85 | Lucidin omega-methyl ether | 284.28 |
| 86 | methyl 6-hydroxy-2,2-dimethyl-3,4-dihydrobenzo[h]chromene-5-carboxylate | 286.35 |
| 87 | rubioncolin B | 562.61 |
| 88 | lucidin primeveroside | 564.54 |
| 89 | Henine | 270.25 |
| 90 | rubiprasin B | 498.87 |
| 91 | Rubilactone | 270.25 |
| 92 | Rubiatriol | 442.8 |
| 93 | Xyloidone | 240.27 |
| 94 | 9-oxo-4-xanthenecarboxylic acid | 240.22 |
| 95 | 4-Hydroxy-3-methoxycinnamaldehyde | 178.2 |
| 96 | Polyphenolic | 302.2 |

Table S2 Go annotation of the expressed drug-disease crossover targets

| ID | Description | GeneRatio | BgRatio | geneID |
| --- | --- | --- | --- | --- |
| GO:0004879 | nuclear receptor activity | 7/75 | 52/18352 | ESR1/AR/PGR/RXRA/PPARG/ESR2/NR3C2 |
| GO:0098531 | ligand-activated transcription factor activity | 7/75 | 52/18352 | ESR1/AR/PGR/RXRA/PPARG/ESR2/NR3C2 |
| GO:0061629 | RNA polymerase II-specific DNA-binding transcription factor binding | 11/75 | 267/18352 | ESR1/RELA/NFKBIA/PRKCB/RXRA/NCOA2/PPARG/MAPK14/GSK3B/JUN/NCOA1 |
| GO:0140297 | DNA-binding transcription factor binding | 12/75 | 347/18352 | ESR1/RELA/NFKBIA/PRKCB/RXRA/NCOA2/BCL2/PPARG/MAPK14/GSK3B/JUN/NCOA1 |
| GO:0042277 | peptide binding | 11/75 | 308/18352 | RELA/NFKBIA/GSTP1/GSTM1/RXRA/ADRB2/GRIA2/OPRD1/ACHE/OPRM1/PPARG |
| GO:0003707 | steroid hormone receptor activity | 5/75 | 26/18352 | ESR1/PGR/RXRA/ESR2/NR3C2 |
| GO:0033218 | amide binding | 11/75 | 381/18352 | RELA/NFKBIA/GSTP1/GSTM1/RXRA/ADRB2/GRIA2/OPRD1/ACHE/OPRM1/PPARG |
| GO:0044389 | ubiquitin-like protein ligase binding | 10/75 | 316/18352 | RELA/CDKN1A/NFKBIA/BCL2/TP53/CASP8/CCNB1/EGFR/GSK3B/JUN |
| GO:0005126 | cytokine receptor binding | 9/75 | 271/18352 | VEGFA/CXCL8/IL10/TNF/IL6/IL1B/CASP3/CASP8/GATA3 |
| GO:0004364 | glutathione transferase activity | 4/75 | 25/18352 | GSTP1/GSTM1/GSTA1/GSTA2 |
| GO:0031625 | ubiquitin protein ligase binding | 9/75 | 297/18352 | RELA/CDKN1A/NFKBIA/BCL2/TP53/CASP8/EGFR/GSK3B/JUN |
| GO:0016922 | nuclear receptor binding | 6/75 | 101/18352 | ESR1/PRKCB/RXRA/NCOA2/PPARG/NCOA1 |
| GO:0008144 | drug binding | 6/75 | 104/18352 | GSTP1/CHRM3/SLC6A4/DRD3/DRD2/PPARG |
| GO:0001223 | transcription coactivator binding | 4/75 | 29/18352 | ESR1/AR/PGR/RELA |
| GO:0030594 | neurotransmitter receptor activity | 6/75 | 111/18352 | CHRM1/CHRM3/GRIA2/HTR3A/DRD3/DRD2 |
| GO:0097199 | cysteine-type endopeptidase activity involved in apoptotic signaling pathway | 3/75 | 10/18352 | CASP9/CASP3/CASP8 |
| GO:0051400 | BH domain binding | 3/75 | 11/18352 | BCL2/BCL2L1/BAX |
| GO:0070513 | death domain binding | 3/75 | 11/18352 | BCL2/BCL2L1/BAX |
| GO:0020037 | heme binding | 6/75 | 138/18352 | PTGS1/PTGS2/HMOX1/CYP1A2/CYP1A1/NOS2 |
| GO:1901338 | catecholamine binding | 3/75 | 14/18352 | ADRB2/DRD3/DRD2 |
| GO:0016209 | antioxidant activity | 5/75 | 86/18352 | GSTP1/GSTA1/PTGS1/PTGS2/NQO1 |
| GO:0005178 | integrin binding | 6/75 | 144/18352 | IGF2/KDR/IL1B/EGFR/ICAM1/PRKCA |
| GO:0035257 | nuclear hormone receptor binding | 6/75 | 144/18352 | ESR1/PRKCB/RXRA/NCOA2/PPARG/NCOA1 |
| GO:0097153 | cysteine-type endopeptidase activity involved in apoptotic process | 3/75 | 15/18352 | CASP9/CASP3/CASP8 |
| GO:0046906 | tetrapyrrole binding | 6/75 | 148/18352 | PTGS1/PTGS2/HMOX1/CYP1A2/CYP1A1/NOS2 |
| GO:0035173 | histone kinase activity | 3/75 | 17/18352 | PRKCB/CCNB1/PRKCA |
| GO:0005125 | cytokine activity | 7/75 | 235/18352 | VEGFA/CXCL8/BMP4/IL10/TNF/IL6/IL1B |
| GO:0016538 | cyclin-dependent protein serine/threonine kinase regulator activity | 4/75 | 50/18352 | CDKN1A/CASP3/CCNB1/CCNA2 |
| GO:0016705 | oxidoreductase activity, acting on paired donors, with incorporation or reduction of molecular oxygen | 6/75 | 162/18352 | PTGS1/PTGS2/HMOX1/CYP1A2/CYP1A1/NOS2 |
| GO:0001221 | transcription cofactor binding | 4/75 | 51/18352 | ESR1/AR/PGR/RELA |
| GO:0004601 | peroxidase activity | 4/75 | 52/18352 | GSTP1/GSTA1/PTGS1/PTGS2 |
| GO:0001091 | RNA polymerase II general transcription initiation factor binding | 3/75 | 19/18352 | ESR1/AR/TP53 |
| GO:0005496 | steroid binding | 5/75 | 106/18352 | ESR1/AR/PGR/ESR2/NR3C2 |
| GO:0001085 | RNA polymerase II transcription factor binding | 4/75 | 56/18352 | AR/TP53/GATA1/GSK3B |
| GO:0016684 | oxidoreductase activity, acting on peroxide as acceptor | 4/75 | 56/18352 | GSTP1/GSTA1/PTGS1/PTGS2 |
| GO:0016765 | transferase activity, transferring alkyl or aryl (other than methyl) groups | 4/75 | 56/18352 | GSTP1/GSTM1/GSTA1/GSTA2 |
| GO:0051427 | hormone receptor binding | 6/75 | 177/18352 | ESR1/PRKCB/RXRA/NCOA2/PPARG/NCOA1 |
| GO:0098960 | postsynaptic neurotransmitter receptor activity | 4/75 | 65/18352 | CHRM1/CHRM3/DRD3/DRD2 |
| GO:0033293 | monocarboxylic acid binding | 4/75 | 72/18352 | GSTP1/GSTA1/RXRA/PPARG |
| GO:0002020 | protease binding | 5/75 | 137/18352 | TNF/BCL2/CASP3/TP53/GSK3B |
| GO:0019207 | kinase regulator activity | 6/75 | 216/18352 | CDKN1A/GSTP1/IGF2/CASP3/CCNB1/CCNA2 |
| GO:0051059 | NF-kappaB binding | 3/75 | 31/18352 | RELA/NFKBIA/GSK3B |
| GO:0070851 | growth factor receptor binding | 5/75 | 141/18352 | VEGFA/IL10/IL6/IL1B/GATA3 |
| GO:0035258 | steroid hormone receptor binding | 4/75 | 81/18352 | ESR1/PRKCB/PPARG/NCOA1 |
| GO:0019903 | protein phosphatase binding | 5/75 | 149/18352 | BCL2/TP53/EGFR/PPARG/MAPK14 |
| GO:0051117 | ATPase binding | 4/75 | 88/18352 | ESR1/AR/PGR/EGFR |
| GO:0008083 | growth factor activity | 5/75 | 162/18352 | VEGFA/IGF2/BMP4/IL10/IL6 |
| GO:0005504 | fatty acid binding | 3/75 | 39/18352 | GSTP1/GSTA1/PPARG |
| GO:0030331 | estrogen receptor binding | 3/75 | 42/18352 | ESR1/PPARG/NCOA1 |
| GO:0099528 | G protein-coupled neurotransmitter receptor activity | 2/75 | 10/18352 | CHRM1/CHRM3 |
| GO:0140296 | general transcription initiation factor binding | 3/75 | 44/18352 | ESR1/AR/TP53 |
| GO:0048018 | receptor ligand activity | 8/75 | 487/18352 | VEGFA/CXCL8/IGF2/BMP4/IL10/TNF/IL6/IL1B |
| GO:0030546 | signaling receptor activator activity | 8/75 | 492/18352 | VEGFA/CXCL8/IGF2/BMP4/IL10/TNF/IL6/IL1B |
| GO:0019887 | protein kinase regulator activity | 5/75 | 185/18352 | CDKN1A/IGF2/CASP3/CCNB1/CCNA2 |
| GO:0032813 | tumor necrosis factor receptor superfamily binding | 3/75 | 48/18352 | TNF/CASP3/CASP8 |
| GO:0004861 | cyclin-dependent protein serine/threonine kinase inhibitor activity | 2/75 | 12/18352 | CDKN1A/CASP3 |
| GO:0043176 | amine binding | 2/75 | 12/18352 | SLC6A4/HTR3A |
| GO:0043295 | glutathione binding | 2/75 | 12/18352 | GSTP1/GSTM1 |
| GO:0051378 | serotonin binding | 2/75 | 12/18352 | SLC6A4/HTR3A |
| GO:0017171 | serine hydrolase activity | 5/75 | 191/18352 | MMP2/MMP9/PRSS3/PREP/ACHE |
| GO:0008227 | G protein-coupled amine receptor activity | 3/75 | 51/18352 | CHRM1/CHRM3/ADRB2 |
| GO:0019902 | phosphatase binding | 5/75 | 194/18352 | BCL2/TP53/EGFR/PPARG/MAPK14 |
| GO:0001162 | RNA polymerase II intronic transcription regulatory region sequence-specific DNA binding | 2/75 | 13/18352 | RXRA/NCOA2 |
| GO:0016653 | oxidoreductase activity, acting on NAD(P)H, heme protein as acceptor | 2/75 | 13/18352 | NQO1/NOS2 |
| GO:0042166 | acetylcholine binding | 2/75 | 13/18352 | CHRM3/ACHE |
| GO:1900750 | oligopeptide binding | 2/75 | 13/18352 | GSTP1/GSTM1 |
| GO:0030374 | nuclear receptor transcription coactivator activity | 3/75 | 54/18352 | PRKCB/NCOA2/NCOA1 |
| GO:0043621 | protein self-association | 3/75 | 57/18352 | TP53/ACHE/PPARG |
| GO:0070742 | C2H2 zinc finger domain binding | 2/75 | 15/18352 | GATA2/GATA1 |
| GO:0031406 | carboxylic acid binding | 5/75 | 212/18352 | GSTP1/GSTA1/RXRA/PPARG/NOS2 |
| GO:0001161 | intronic transcription regulatory region sequence-specific DNA binding | 2/75 | 16/18352 | RXRA/NCOA2 |
| GO:0004697 | protein kinase C activity | 2/75 | 16/18352 | PRKCB/PRKCA |
| GO:0004698 | calcium-dependent protein kinase C activity | 2/75 | 16/18352 | PRKCB/PRKCA |
| GO:0071837 | HMG box domain binding | 2/75 | 16/18352 | GATA3/JUN |
| GO:0004175 | endopeptidase activity | 7/75 | 440/18352 | MMP2/MMP9/PRSS3/PREP/CASP9/CASP3/CASP8 |
| GO:0043177 | organic acid binding | 5/75 | 224/18352 | GSTP1/GSTA1/RXRA/PPARG/NOS2 |
| GO:0046965 | retinoid X receptor binding | 2/75 | 18/18352 | PPARG/NCOA1 |
| GO:0002039 | p53 binding | 3/75 | 66/18352 | TP53/GATA1/GSK3B |
| GO:0005123 | death receptor binding | 2/75 | 20/18352 | CASP3/CASP8 |
| GO:0016247 | channel regulator activity | 4/75 | 148/18352 | PRKCB/TRPV1/ADRB2/BCL2 |
| GO:0001098 | basal transcription machinery binding | 3/75 | 72/18352 | ESR1/AR/TP53 |
| GO:0001099 | basal RNA polymerase II transcription machinery binding | 3/75 | 72/18352 | ESR1/AR/TP53 |
| GO:0004602 | glutathione peroxidase activity | 2/75 | 21/18352 | GSTP1/GSTA1 |
| GO:0042165 | neurotransmitter binding | 2/75 | 21/18352 | CHRM3/ACHE |
| GO:0005230 | extracellular ligand-gated ion channel activity | 3/75 | 74/18352 | TRPV1/GRIA2/HTR3A |
| GO:0070491 | repressing transcription factor binding | 3/75 | 74/18352 | RELA/BCL2/PPARG |
| GO:0015464 | acetylcholine receptor activity | 2/75 | 22/18352 | CHRM1/CHRM3 |
| GO:0001540 | amyloid-beta binding | 3/75 | 77/18352 | ADRB2/GRIA2/ACHE |
| GO:0009931 | calcium-dependent protein serine/threonine kinase activity | 2/75 | 23/18352 | PRKCB/PRKCA |
| GO:0010857 | calcium-dependent protein kinase activity | 2/75 | 24/18352 | PRKCB/PRKCA |
| GO:0033613 | activating transcription factor binding | 3/75 | 80/18352 | RELA/PPARG/JUN |
| GO:0030295 | protein kinase activator activity | 3/75 | 82/18352 | CDKN1A/IGF2/CCNB1 |
| GO:0070330 | aromatase activity | 2/75 | 25/18352 | CYP1A2/CYP1A1 |
| GO:0004435 | phosphatidylinositol phospholipase C activity | 2/75 | 26/18352 | CHRM1/CHRM3 |
| GO:0042923 | neuropeptide binding | 2/75 | 26/18352 | OPRD1/OPRM1 |
| GO:0004252 | serine-type endopeptidase activity | 4/75 | 169/18352 | MMP2/MMP9/PRSS3/PREP |
| GO:0008013 | beta-catenin binding | 3/75 | 85/18352 | ESR1/AR/GSK3B |
| GO:0042974 | retinoic acid receptor binding | 2/75 | 27/18352 | PPARG/NCOA1 |
| GO:0008081 | phosphoric diester hydrolase activity | 3/75 | 89/18352 | CHRM1/CHRM3/HMOX1 |
| GO:0019209 | kinase activator activity | 3/75 | 89/18352 | CDKN1A/IGF2/CCNB1 |
| GO:0004629 | phospholipase C activity | 2/75 | 28/18352 | CHRM1/CHRM3 |
| GO:0033612 | receptor serine/threonine kinase binding | 2/75 | 29/18352 | BMP4/OPRD1 |
| GO:0005164 | tumor necrosis factor receptor binding | 2/75 | 31/18352 | TNF/CASP8 |
| GO:0030291 | protein serine/threonine kinase inhibitor activity | 2/75 | 31/18352 | CDKN1A/CASP3 |
| GO:0008236 | serine-type peptidase activity | 4/75 | 187/18352 | MMP2/MMP9/PRSS3/PREP |
| GO:0051721 | protein phosphatase 2A binding | 2/75 | 32/18352 | BCL2/TP53 |
| GO:0004497 | monooxygenase activity | 3/75 | 101/18352 | CYP1A2/CYP1A1/NOS2 |
| GO:0004993 | G protein-coupled serotonin receptor activity | 2/75 | 34/18352 | CHRM1/CHRM3 |
| GO:0099589 | serotonin receptor activity | 2/75 | 34/18352 | CHRM1/CHRM3 |
| GO:0016712 | oxidoreductase activity, acting on paired donors, with incorporation or reduction of molecular oxygen, reduced flavin or flavoprotein as one donor, and incorporation of one atom of oxygen | 2/75 | 35/18352 | CYP1A2/CYP1A1 |
| GO:0005516 | calmodulin binding | 4/75 | 200/18352 | TRPV1/EGFR/MAP2/NOS2 |
| GO:0004620 | phospholipase activity | 3/75 | 105/18352 | CHRM1/CHRM3/HMOX1 |
| GO:0031490 | chromatin DNA binding | 3/75 | 106/18352 | RELA/RXRA/GATA1 |
| GO:0042805 | actinin binding | 2/75 | 36/18352 | RELA/PPARG |
| GO:0047485 | protein N-terminus binding | 3/75 | 107/18352 | RELA/TP53/NCOA1 |
| GO:0099094 | ligand-gated cation channel activity | 3/75 | 108/18352 | TRPV1/GRIA2/HTR3A |
| GO:0042056 | chemoattractant activity | 2/75 | 37/18352 | VEGFA/BMP4 |
| GO:0008395 | steroid hydroxylase activity | 2/75 | 38/18352 | CYP1A2/CYP1A1 |
| GO:0032451 | demethylase activity | 2/75 | 38/18352 | CYP1A2/CYP1A1 |
| GO:0043539 | protein serine/threonine kinase activator activity | 2/75 | 38/18352 | IGF2/CCNB1 |
| GO:0004197 | cysteine-type endopeptidase activity | 3/75 | 114/18352 | CASP9/CASP3/CASP8 |
| GO:0001227 | DNA-binding transcription repressor activity, RNA polymerase II-specific | 5/75 | 335/18352 | RELA/TBX21/GATA3/PPARG/JUN |
| GO:0001217 | DNA-binding transcription repressor activity | 5/75 | 336/18352 | RELA/TBX21/GATA3/PPARG/JUN |
| GO:0030544 | Hsp70 protein binding | 2/75 | 42/18352 | BAX/CYP1A1 |
| GO:0051879 | Hsp90 protein binding | 2/75 | 42/18352 | KDR/CYP1A1 |
| GO:0015267 | channel activity | 6/75 | 479/18352 | TRPV1/GRIA2/BCL2/BAX/HTR3A/OPRM1 |
| GO:0022803 | passive transmembrane transporter activity | 6/75 | 480/18352 | TRPV1/GRIA2/BCL2/BAX/HTR3A/OPRM1 |
| GO:0016709 | oxidoreductase activity, acting on paired donors, with incorporation or reduction of molecular oxygen, NAD(P)H as one donor, and incorporation of one atom of oxygen | 2/75 | 45/18352 | CYP1A1/NOS2 |
| GO:0048156 | tau protein binding | 2/75 | 45/18352 | GSK3B/MAP2 |
| GO:0001046 | core promoter sequence-specific DNA binding | 2/75 | 46/18352 | RELA/TP53 |
| GO:0035254 | glutamate receptor binding | 2/75 | 46/18352 | RASGRF1/DRD2 |
| GO:0031072 | heat shock protein binding | 3/75 | 127/18352 | KDR/BAX/CYP1A1 |
| GO:0016298 | lipase activity | 3/75 | 130/18352 | CHRM1/CHRM3/HMOX1 |
| GO:0070888 | E-box binding | 2/75 | 50/18352 | GATA3/PPARG |
| GO:0008234 | cysteine-type peptidase activity | 3/75 | 139/18352 | CASP9/CASP3/CASP8 |
| GO:0015276 | ligand-gated ion channel activity | 3/75 | 139/18352 | TRPV1/GRIA2/HTR3A |
| GO:0022834 | ligand-gated channel activity | 3/75 | 139/18352 | TRPV1/GRIA2/HTR3A |

Table S3 KEGG enrichment analysis

| ID | Description | BgRatio | geneID |
| --- | --- | --- | --- |
| hsa05418 | Fluid shear stress and atherosclerosis | 139/8109 | RELA/VEGFA/MMP2/MMP9/GSTP1/GSTM1/GSTA1/GSTA2/KDR/HMOX1/PECAM1/BMP4/TNF/IL1B/BCL2/TP53/ICAM1/NQO1/MAPK14/JUN |
| hsa05417 | Lipid and atherosclerosis | 215/8109 | RELA/MMP9/NFKBIA/CXCL8/RXRA/TNF/IL6/IL1B/BCL2/BCL2L1/BAX/CASP9/CASP3/TP53/CASP8/CYP1A1/ICAM1/PPARG/MAPK14/GSK3B/JUN/PRKCA |
| hsa05161 | Hepatitis B | 162/8109 | RELA/CDKN1A/MMP9/NFKBIA/CXCL8/PRKCB/TNF/IL6/BCL2/BAX/CASP9/CASP3/TP53/CASP8/MAPK14/CCNA2/JUN/PRKCA |
| hsa04933 | AGE-RAGE signaling pathway in diabetic complications | 100/8109 | RELA/VEGFA/MMP2/CXCL8/PRKCB/TNF/IL6/IL1B/BCL2/BAX/CASP3/ICAM1/MAPK14/JUN/PRKCA |
| hsa05163 | Human cytomegalovirus infection | 225/8109 | RELA/VEGFA/CDKN1A/NFKBIA/CXCL8/PRKCB/PTGS2/TNF/IL6/IL1B/BAX/CASP9/CASP3/TP53/CASP8/EGFR/MAPK14/GSK3B/PRKCA |
| hsa05207 | Chemical carcinogenesis - receptor activation | 212/8109 | ESR1/AR/PGR/RELA/VEGFA/PRKCB/GSTM1/GSTA1/GSTA2/RXRA/ADRB2/BCL2/CYP1A2/CYP1A1/EGFR/ESR2/JUN/PRKCA |
| hsa01524 | Platinum drug resistance | 73/8109 | CDKN1A/GSTP1/GSTM1/GSTA1/GSTA2/BCL2/BCL2L1/BAX/CASP9/CASP3/TP53/CASP8 |
| hsa04657 | IL-17 signaling pathway | 94/8109 | RELA/MMP9/NFKBIA/CXCL8/PTGS2/TNF/IL6/IL1B/CASP3/CASP8/MAPK14/GSK3B/JUN |
| hsa05167 | Kaposi sarcoma-associated herpesvirus infection | 194/8109 | RELA/VEGFA/CDKN1A/NFKBIA/CXCL8/PTGS2/IL6/BAX/CASP9/CASP3/TP53/CASP8/ICAM1/MAPK14/GSK3B/JUN |
| hsa05225 | Hepatocellular carcinoma | 168/8109 | CDKN1A/PRKCB/GSTP1/IGF2/GSTM1/GSTA1/GSTA2/HMOX1/BCL2L1/BAX/TP53/EGFR/NQO1/GSK3B/PRKCA |
| hsa05222 | Small cell lung cancer | 92/8109 | RELA/CDKN1A/NFKBIA/PTGS2/RXRA/BCL2/BCL2L1/BAX/CASP9/CASP3/TP53/NOS2 |
| hsa05169 | Epstein-Barr virus infection | 202/8109 | RELA/CDKN1A/NFKBIA/TNF/IL6/BCL2/BAX/CASP9/CASP3/TP53/CASP8/ICAM1/MAPK14/CCNA2/JUN |
| hsa05162 | Measles | 139/8109 | RELA/NFKBIA/IL6/IL1B/BCL2/BCL2L1/BAX/CASP9/CASP3/TP53/CASP8/GSK3B/JUN |
| hsa05164 | Influenza A | 171/8109 | RELA/NFKBIA/CXCL8/PRKCB/PRSS3/TNF/IL6/IL1B/BAX/CASP9/CASP3/CASP8/ICAM1/PRKCA |
| hsa04668 | TNF signaling pathway | 112/8109 | RELA/MMP9/NFKBIA/PTGS2/TNF/IL6/IL1B/CASP3/CASP8/ICAM1/MAPK14/JUN |
| hsa05215 | Prostate cancer | 97/8109 | AR/RELA/CDKN1A/MMP9/NFKBIA/GSTP1/BCL2/CASP9/TP53/EGFR/GSK3B |
| hsa04932 | Non-alcoholic fatty liver disease | 155/8109 | RELA/CXCL8/RXRA/TNF/IL6/IL1B/BAX/CASP3/CASP8/PPARG/MAPK14/GSK3B/JUN |
| hsa01522 | Endocrine resistance | 98/8109 | ESR1/CDKN1A/MMP2/MMP9/BCL2/BAX/TP53/EGFR/ESR2/MAPK14/JUN |
| hsa05133 | Pertussis | 76/8109 | RELA/CXCL8/IL10/TNF/IL6/IL1B/CASP3/MAPK14/JUN/NOS2 |
| hsa05140 | Leishmaniasis | 77/8109 | RELA/NFKBIA/PRKCB/PTGS2/IL10/TNF/IL1B/MAPK14/JUN/NOS2 |
| hsa05142 | Chagas disease | 102/8109 | RELA/NFKBIA/CXCL8/IL10/TNF/IL6/IL1B/CASP8/MAPK14/JUN/NOS2 |
| hsa05134 | Legionellosis | 57/8109 | RELA/NFKBIA/CXCL8/TNF/IL6/IL1B/CASP9/CASP3/CASP8 |
| hsa01521 | EGFR tyrosine kinase inhibitor resistance | 79/8109 | VEGFA/PRKCB/KDR/IL6/BCL2/BCL2L1/BAX/EGFR/GSK3B/PRKCA |
| hsa05205 | Proteoglycans in cancer | 205/8109 | ESR1/VEGFA/CDKN1A/MMP2/MMP9/PRKCB/IGF2/KDR/TNF/CASP3/TP53/EGFR/MAPK14/PRKCA |
| hsa05145 | Toxoplasmosis | 112/8109 | RELA/NFKBIA/IL10/TNF/BCL2/BCL2L1/CASP9/CASP3/CASP8/MAPK14/NOS2 |
| hsa05170 | Human immunodeficiency virus 1 infection | 212/8109 | RELA/NFKBIA/PRKCB/TNF/BCL2/BCL2L1/BAX/CASP9/CASP3/CASP8/CCNB1/MAPK14/JUN/PRKCA |
| hsa05160 | Hepatitis C | 157/8109 | RELA/CDKN1A/NFKBIA/RXRA/TNF/BAX/CASP9/CASP3/TP53/CASP8/EGFR/GSK3B |
| hsa04115 | p53 signaling pathway | 73/8109 | CDKN1A/BCL2/BCL2L1/BAX/CASP9/CASP3/TP53/CASP8/CCNB1 |
| hsa05022 | Pathways of neurodegeneration - multiple diseases | 476/8109 | RELA/PRKCB/PTGS2/CHRM1/CHRM3/GRIA2/TNF/IL6/IL1B/BCL2/BCL2L1/BAX/CASP9/CASP3/CASP8/MAPK14/GSK3B/PRKCA/NOS2 |
| hsa05146 | Amoebiasis | 102/8109 | RELA/CXCL8/PRKCB/IL10/TNF/IL6/IL1B/CASP3/PRKCA/NOS2 |
| hsa04064 | NF-kappa B signaling pathway | 104/8109 | RELA/NFKBIA/CXCL8/PRKCB/PTGS2/TNF/IL1B/BCL2/BCL2L1/ICAM1 |
| hsa04625 | C-type lectin receptor signaling pathway | 104/8109 | RELA/NFKBIA/PTGS2/IL10/TNF/IL6/IL1B/CASP8/MAPK14/JUN |
| hsa04210 | Apoptosis | 136/8109 | RELA/NFKBIA/TNF/BCL2/BCL2L1/BAX/CASP9/CASP3/TP53/CASP8/JUN |
| hsa04915 | Estrogen signaling pathway | 138/8109 | ESR1/PGR/MMP2/MMP9/NCOA2/BCL2/OPRM1/EGFR/ESR2/JUN/NCOA1 |
| hsa05143 | African trypanosomiasis | 37/8109 | PRKCB/IL10/TNF/IL6/IL1B/ICAM1/PRKCA |
| hsa04066 | HIF-1 signaling pathway | 109/8109 | RELA/VEGFA/CDKN1A/PRKCB/HMOX1/IL6/BCL2/EGFR/PRKCA/NOS2 |
| hsa05152 | Tuberculosis | 180/8109 | RELA/IL10/TNF/IL6/IL1B/BCL2/BAX/CASP9/CASP3/CASP8/MAPK14/NOS2 |
| hsa05210 | Colorectal cancer | 86/8109 | CDKN1A/BCL2/BAX/CASP9/CASP3/TP53/EGFR/GSK3B/JUN |
| hsa05208 | Chemical carcinogenesis - reactive oxygen species | 223/8109 | RELA/VEGFA/NFKBIA/GSTM1/GSTA1/GSTA2/HMOX1/CYP1A2/CYP1A1/EGFR/NQO1/MAPK14/JUN |
| hsa05219 | Bladder cancer | 41/8109 | VEGFA/CDKN1A/MMP2/MMP9/CXCL8/TP53/EGFR |
| hsa05321 | Inflammatory bowel disease | 65/8109 | RELA/TBX21/IL10/TNF/IL6/IL1B/GATA3/JUN |
| hsa04919 | Thyroid hormone signaling pathway | 121/8109 | ESR1/PRKCB/RXRA/NCOA2/BMP4/CASP9/TP53/GSK3B/PRKCA/NCOA1 |
| hsa05130 | Pathogenic Escherichia coli infection | 197/8109 | RELA/NFKBIA/CXCL8/TNF/IL6/IL1B/BAX/CASP9/CASP3/CASP8/MAPK14/JUN |
| hsa04926 | Relaxin signaling pathway | 129/8109 | RELA/VEGFA/MMP2/MMP9/NFKBIA/EGFR/MAPK14/JUN/PRKCA/NOS2 |
| hsa05131 | Shigellosis | 247/8109 | RELA/NFKBIA/CXCL8/TNF/IL1B/BCL2/BCL2L1/BAX/TP53/EGFR/MAPK14/GSK3B/JUN |
| hsa04010 | MAPK signaling pathway | 294/8109 | RELA/VEGFA/PRKCB/IGF2/KDR/TNF/IL1B/CASP3/TP53/RASGRF1/EGFR/MAPK14/JUN/PRKCA |
| hsa05223 | Non-small cell lung cancer | 72/8109 | CDKN1A/PRKCB/RXRA/BAX/CASP9/TP53/EGFR/PRKCA |
| hsa05144 | Malaria | 50/8109 | CXCL8/PECAM1/IL10/TNF/IL6/IL1B/ICAM1 |
| hsa04620 | Toll-like receptor signaling pathway | 104/8109 | RELA/NFKBIA/CXCL8/TNF/IL6/IL1B/CASP8/MAPK14/JUN |
| hsa05135 | Yersinia infection | 137/8109 | RELA/NFKBIA/CXCL8/IL10/TNF/IL6/IL1B/MAPK14/GSK3B/JUN |
| hsa04151 | PI3K-Akt signaling pathway | 354/8109 | RELA/VEGFA/CDKN1A/IGF2/RXRA/CHRM1/KDR/IL6/BCL2/BCL2L1/CASP9/TP53/EGFR/GSK3B/PRKCA |
| hsa05212 | Pancreatic cancer | 76/8109 | RELA/VEGFA/CDKN1A/BCL2L1/BAX/CASP9/TP53/EGFR |
| hsa04215 | Apoptosis - multiple species | 32/8109 | BCL2/BCL2L1/BAX/CASP9/CASP3/CASP8 |
| hsa04659 | Th17 cell differentiation | 108/8109 | RELA/NFKBIA/TBX21/RXRA/IL6/IL1B/GATA3/MAPK14/JUN |
| hsa04936 | Alcoholic liver disease | 142/8109 | RELA/NFKBIA/CXCL8/TNF/IL6/IL1B/CASP3/CASP8/MAPK14/GSK3B |
| hsa04621 | NOD-like receptor signaling pathway | 184/8109 | RELA/NFKBIA/CXCL8/TNF/IL6/IL1B/BCL2/BCL2L1/CASP8/MAPK14/JUN |
| hsa05224 | Breast cancer | 147/8109 | ESR1/PGR/CDKN1A/BAX/TP53/EGFR/ESR2/GSK3B/JUN/NCOA1 |
| hsa04370 | VEGF signaling pathway | 59/8109 | VEGFA/PRKCB/PTGS2/KDR/CASP9/MAPK14/PRKCA |
| hsa05202 | Transcriptional misregulation in cancer | 192/8109 | RELA/CDKN1A/MMP9/CXCL8/RXRA/IL6/BCL2L1/BAX/TP53/PPARG/CCNA2 |
| hsa04071 | Sphingolipid signaling pathway | 119/8109 | RELA/PRKCB/TNF/BCL2/BAX/TP53/OPRD1/MAPK14/PRKCA |
| hsa05132 | Salmonella infection | 249/8109 | RELA/NFKBIA/CXCL8/TNF/IL6/IL1B/BCL2/BAX/CASP3/CASP8/MAPK14/JUN |
| hsa05204 | Chemical carcinogenesis - DNA adducts | 69/8109 | GSTP1/GSTM1/GSTA1/GSTA2/PTGS2/CYP1A2/CYP1A1 |
| hsa05120 | Epithelial cell signaling in Helicobacter pylori infection | 70/8109 | RELA/NFKBIA/CXCL8/CASP3/EGFR/MAPK14/JUN |
| hsa05166 | Human T-cell leukemia virus 1 infection | 222/8109 | RELA/CDKN1A/NFKBIA/TNF/IL6/BCL2L1/BAX/TP53/ICAM1/CCNA2/JUN |
| hsa05171 | Coronavirus disease - COVID-19 | 232/8109 | RELA/NFKBIA/CXCL8/PRKCB/TNF/IL6/IL1B/EGFR/MAPK14/JUN/PRKCA |
| hsa04722 | Neurotrophin signaling pathway | 119/8109 | RELA/NFKBIA/BCL2/BAX/TP53/MAPK14/GSK3B/JUN |
| hsa05213 | Endometrial cancer | 58/8109 | CDKN1A/BAX/CASP9/TP53/EGFR/GSK3B |
| hsa05323 | Rheumatoid arthritis | 93/8109 | VEGFA/CXCL8/TNF/IL6/IL1B/ICAM1/JUN |
| hsa05216 | Thyroid cancer | 37/8109 | CDKN1A/RXRA/BAX/TP53/PPARG |
| hsa05165 | Human papillomavirus infection | 331/8109 | RELA/VEGFA/CDKN1A/PTGS2/TNF/BAX/CASP3/TP53/CASP8/EGFR/GSK3B/CCNA2 |
| hsa04622 | RIG-I-like receptor signaling pathway | 70/8109 | RELA/NFKBIA/CXCL8/TNF/CASP8/MAPK14 |
| hsa04660 | T cell receptor signaling pathway | 104/8109 | RELA/NFKBIA/IL10/TNF/MAPK14/GSK3B/JUN |
| hsa04928 | Parathyroid hormone synthesis, secretion and action | 106/8109 | CDKN1A/PRKCB/RXRA/BCL2/EGFR/GATA3/PRKCA |
| hsa05214 | Glioma | 75/8109 | CDKN1A/PRKCB/BAX/TP53/EGFR/PRKCA |
| hsa05220 | Chronic myeloid leukemia | 76/8109 | RELA/CDKN1A/NFKBIA/BCL2L1/BAX/TP53 |
| hsa04510 | Focal adhesion | 201/8109 | VEGFA/PRKCB/KDR/BCL2/RASGRF1/EGFR/GSK3B/JUN/PRKCA |
| hsa04670 | Leukocyte transendothelial migration | 114/8109 | MMP2/MMP9/PRKCB/PECAM1/ICAM1/MAPK14/PRKCA |
| hsa00980 | Metabolism of xenobiotics by cytochrome P450 | 78/8109 | GSTP1/GSTM1/GSTA1/GSTA2/CYP1A2/CYP1A1 |
| hsa04218 | Cellular senescence | 156/8109 | RELA/CDKN1A/CXCL8/IL6/TP53/CCNB1/MAPK14/CCNA2 |
| hsa04726 | Serotonergic synapse | 115/8109 | PRKCB/PTGS1/PTGS2/SLC6A4/CASP3/HTR3A/PRKCA |
| hsa05203 | Viral carcinogenesis | 204/8109 | RELA/CDKN1A/NFKBIA/BAX/CASP3/TP53/CASP8/CCNA2/JUN |
| hsa05206 | MicroRNAs in cancer | 310/8109 | VEGFA/CDKN1A/MMP9/PRKCB/PTGS2/HMOX1/BCL2/CASP3/TP53/EGFR/PRKCA |
| hsa04012 | ErbB signaling pathway | 85/8109 | CDKN1A/PRKCB/EGFR/GSK3B/JUN/PRKCA |
| hsa05010 | Alzheimer disease | 384/8109 | RELA/PTGS2/CHRM1/CHRM3/TNF/IL6/IL1B/CASP9/CASP3/CASP8/GSK3B/NOS2 |
| hsa04380 | Osteoclast differentiation | 128/8109 | RELA/NFKBIA/TNF/IL1B/PPARG/MAPK14/JUN |
| hsa04728 | Dopaminergic synapse | 132/8109 | PRKCB/GRIA2/DRD3/DRD2/MAPK14/GSK3B/PRKCA |
| hsa04658 | Th1 and Th2 cell differentiation | 92/8109 | RELA/NFKBIA/TBX21/GATA3/MAPK14/JUN |
| hsa01523 | Antifolate resistance | 31/8109 | RELA/TNF/IL6/IL1B |
| hsa04912 | GnRH signaling pathway | 93/8109 | MMP2/PRKCB/EGFR/MAPK14/JUN/PRKCA |
| hsa04014 | Ras signaling pathway | 232/8109 | RELA/VEGFA/PRKCB/IGF2/KDR/BCL2L1/RASGRF1/EGFR/PRKCA |
| hsa05217 | Basal cell carcinoma | 63/8109 | CDKN1A/BMP4/BAX/TP53/GSK3B |
| hsa04020 | Calcium signaling pathway | 240/8109 | VEGFA/PRKCB/CHRM1/CHRM3/ADRB2/KDR/EGFR/PRKCA/NOS2 |
| hsa05226 | Gastric cancer | 149/8109 | CDKN1A/RXRA/BCL2/BAX/TP53/EGFR/GSK3B |
| hsa04931 | Insulin resistance | 108/8109 | RELA/NFKBIA/PRKCB/TNF/IL6/GSK3B |
| hsa04917 | Prolactin signaling pathway | 70/8109 | ESR1/RELA/ESR2/MAPK14/GSK3B |
| hsa00982 | Drug metabolism - cytochrome P450 | 72/8109 | GSTP1/GSTM1/GSTA1/GSTA2/CYP1A2 |
| hsa04725 | Cholinergic synapse | 113/8109 | PRKCB/CHRM1/CHRM3/BCL2/ACHE/PRKCA |
| hsa04662 | B cell receptor signaling pathway | 82/8109 | RELA/NFKBIA/PRKCB/GSK3B/JUN |
| hsa05030 | Cocaine addiction | 49/8109 | RELA/GRIA2/DRD2/JUN |
| hsa04080 | Neuroactive ligand-receptor interaction | 353/8109 | TRPV1/CHRM1/PRSS3/CHRM3/ADRB2/GRIA2/OPRD1/OPRM1/DRD3/DRD2 |
| hsa04068 | FoxO signaling pathway | 131/8109 | CDKN1A/IL10/IL6/CCNB1/EGFR/MAPK14 |
| hsa05168 | Herpes simplex virus 1 infection | 495/8109 | RELA/NFKBIA/TNF/IL6/IL1B/BCL2/BCL2L1/BAX/CASP9/CASP3/TP53/CASP8 |
| hsa05235 | PD-L1 expression and PD-1 checkpoint pathway in cancer | 89/8109 | RELA/NFKBIA/EGFR/MAPK14/JUN |
| hsa05014 | Amyotrophic lateral sclerosis | 364/8109 | GRIA2/TNF/BCL2/BCL2L1/BAX/CASP9/CASP3/TP53/MAPK14/NOS2 |
| hsa04970 | Salivary secretion | 93/8109 | PRKCB/CHRM3/ADRB2/AMY2A/PRKCA |
| hsa04750 | Inflammatory mediator regulation of TRP channels | 98/8109 | PRKCB/TRPV1/IL1B/MAPK14/PRKCA |
| hsa00480 | Glutathione metabolism | 58/8109 | GSTP1/GSTM1/GSTA1/GSTA2 |
| hsa05416 | Viral myocarditis | 60/8109 | CASP9/CASP3/CASP8/ICAM1 |
| hsa05415 | Diabetic cardiomyopathy | 203/8109 | RELA/MMP2/MMP9/PRKCB/MAPK14/GSK3B/PRKCA |
| hsa04972 | Pancreatic secretion | 102/8109 | PRKCB/PRSS3/CHRM3/AMY2A/PRKCA |
| hsa04921 | Oxytocin signaling pathway | 154/8109 | CDKN1A/PRKCB/PTGS2/EGFR/JUN/PRKCA |
| hsa04623 | Cytosolic DNA-sensing pathway | 63/8109 | RELA/NFKBIA/IL6/IL1B |
| hsa04015 | Rap1 signaling pathway | 210/8109 | VEGFA/PRKCB/KDR/DRD2/EGFR/MAPK14/PRKCA |
| hsa05020 | Prion disease | 273/8109 | TNF/IL6/IL1B/BAX/CASP9/CASP3/MAPK14/GSK3B |
| hsa04630 | JAK-STAT signaling pathway | 162/8109 | CDKN1A/IL10/IL6/BCL2/BCL2L1/EGFR |
| hsa04024 | cAMP signaling pathway | 221/8109 | RELA/NFKBIA/CHRM1/ADRB2/GRIA2/DRD2/JUN |
| hsa04920 | Adipocytokine signaling pathway | 69/8109 | RELA/NFKBIA/RXRA/TNF |
| hsa05031 | Amphetamine addiction | 69/8109 | PRKCB/GRIA2/JUN/PRKCA |
| hsa04137 | Mitophagy - animal | 72/8109 | RELA/BCL2L1/TP53/JUN |
| hsa05218 | Melanoma | 72/8109 | CDKN1A/BAX/TP53/EGFR |
| hsa04960 | Aldosterone-regulated sodium reabsorption | 37/8109 | PRKCB/PRKCA/NR3C2 |
| hsa04110 | Cell cycle | 126/8109 | CDKN1A/TP53/CCNB1/GSK3B/CCNA2 |
| hsa00983 | Drug metabolism - other enzymes | 80/8109 | GSTP1/GSTM1/GSTA1/GSTA2 |
| hsa04650 | Natural killer cell mediated cytotoxicity | 131/8109 | PRKCB/TNF/CASP3/ICAM1/PRKCA |
| hsa05332 | Graft-versus-host disease | 42/8109 | TNF/IL6/IL1B |
| hsa04540 | Gap junction | 88/8109 | PRKCB/DRD2/EGFR/PRKCA |
| hsa04211 | Longevity regulating pathway | 89/8109 | RELA/BAX/TP53/PPARG |
| hsa04723 | Retrograde endocannabinoid signaling | 148/8109 | PRKCB/PTGS2/GRIA2/MAPK14/PRKCA |
| hsa05231 | Choline metabolism in cancer | 98/8109 | PRKCB/EGFR/JUN/PRKCA |
| hsa04961 | Endocrine and other factor-regulated calcium reabsorption | 53/8109 | ESR1/PRKCB/PRKCA |
| hsa04061 | Viral protein interaction with cytokine and cytokine receptor | 100/8109 | CXCL8/IL10/TNF/IL6 |
| hsa04914 | Progesterone-mediated oocyte maturation | 102/8109 | PGR/CCNB1/MAPK14/CCNA2 |
| hsa04217 | Necroptosis | 159/8109 | TNF/IL1B/BCL2/BAX/CASP8 |
| hsa04923 | Regulation of lipolysis in adipocytes | 56/8109 | PTGS1/PTGS2/ADRB2 |
| hsa04310 | Wnt signaling pathway | 167/8109 | PRKCB/TP53/GSK3B/JUN/PRKCA |
| hsa04730 | Long-term depression | 60/8109 | PRKCB/GRIA2/PRKCA |
| hsa05016 | Huntington disease | 306/8109 | GRIA2/BAX/CASP9/CASP3/TP53/CASP8/PPARG |
| hsa04929 | GnRH secretion | 64/8109 | PRKCB/ESR2/PRKCA |
| hsa04935 | Growth hormone synthesis, secretion and action | 119/8109 | PRKCB/MAPK14/GSK3B/PRKCA |
| hsa04720 | Long-term potentiation | 67/8109 | PRKCB/GRIA2/PRKCA |
| hsa04664 | Fc epsilon RI signaling pathway | 68/8109 | TNF/MAPK14/PRKCA |
| hsa05211 | Renal cell carcinoma | 69/8109 | VEGFA/CDKN1A/JUN |
| hsa04062 | Chemokine signaling pathway | 192/8109 | RELA/NFKBIA/CXCL8/PRKCB/GSK3B |
| hsa04114 | Oocyte meiosis | 131/8109 | AR/PGR/CCNB1/MAPK14 |
| hsa05012 | Parkinson disease | 266/8109 | BCL2L1/BAX/CASP9/CASP3/TP53/DRD2 |
| hsa04971 | Gastric acid secretion | 76/8109 | PRKCB/CHRM3/PRKCA |
| hsa05310 | Asthma | 31/8109 | IL10/TNF |
